# Biotechnological Approach to Evaluate the Immunomodulatory Activity of *Ethanolic Extract* of Tinospora cordifolia Stem (Mango Plant Climber)

**Published:** 2012

**Authors:** Vaibhav Aher, Arun Kumar Wahi

**Affiliations:** a *Institute of Pharmacy, NIMS University, Jaipur,303121, India.*; b *College of Pharmacy, MIT, Moradabad-244001, India.*

**Keywords:** *Tinospora cordifolia *(*Tc*), Immunomodulator, Antioxidant enzymes, Lymphocyte proliferation, Real time PCR

## Abstract

The present study was designed to investigate the immunomodulatory activity of the ethanolic extract of *Tinospora cordifolia *(Family: Menispermaceae) stem (climbing shrub, mango plant) at cellular level. For antioxidant study, the liver mitochondria were separated and the concentration of enzymes like lipid peroxidation (LPO), reduced glutathione (GSH), catalase (CAT) and superoxide Dismutase (SOD) were estimated; melatonin secretion characterization was carried out through SDS-PAGE. The spleen lymphocyte proliferation assay was performed through measuring its optical density at 570 nm using Elisa Reader. The cytokines viz. IL-2, IL-10 and TNF-α expression in spleen cells were determined through Real Time PCR. *Tinospora cordifolia *(*Tc*) ethanolic extract (100 mg/Kg/p.o.) increased the level of liver mitochondrial enzymes like GSH, CAT and SOD but decreased the level of LPO in liver as compared to the vehicle, SRBC and cyclophosphamide-treated groups. The secretion of melatonin via pineal gland was enhanced with *Tc *treatment. The extract also increased the spleen lymphocyte proliferation. In RT-PCR analysis, the expression of cytokines viz. IL-2, IL-10 and TNF-*α *was more in *Tc*-treated animals than vehicle and cyclophosphamide treatment. Hence, the study confirms the immunomodulatory activity of *Tc *stem through altering the concentration of antioxidant enzymes, increasing T and B cells and antibody which play an important role in immunity, enhancing the concentration of melatonin in pineal gland and increasing the level of cytokines like IL-2, IL-10 and TNF-*α *which plays an important role in immunity.

## Introduction

In recent times, focus on plant research has been intensified all over the world and large amount of evidence has been collected to show the immense potential of medicinal plant used in various traditional systems (1). The immune system is involved in the etiology as well as pathophysiologic mechanism of many diseases (2). The modulation of immune responses to alleviate the diseases has been of interest for many years and the concept of ‘Rasayana’ in Ayurveda is based on the related principles (3). Further immunomodulation using medicinal plants can provide an alternative to conventional chemotherapy for a variety of diseases especially when the host defense mechanism has to be acquired under the conditions of impaired immune responsiveness (4). Indian medicinal plants are rich sources of substances which are claimed to induce para-immunity, the non-specific immunomodulation of especially granulocytes, macro-phages, natural killer cells and competent functions (5).

Among the suppressive synthetic substances, cyclophosphamide has been extensively studied (6). However, the major drawback of this drug is myelosuppression, which is undesirable (7). Immunostimulation and immunosuppression both need to be tackled in order to regulate the normal immunological functioning. Therefore, the stimulatory or suppressive agents have been shown to possess activity to normalize or modulate the pathophysiological processes and are hence called immunomodulatory agents (8).

In recent years, immunostimulatory activity has been reported in a number of Ayurvedic plants like *Terminalia chebula *(9, 10), *Curcuma longa *(11), *Lawsonia alba *(12), and *Tinospora cordifolia *(5, 13-16). *Tc *stem is stomachic, diuretic (17), cures jaundice, skin diseases (18), *etc*.

*Tinospora cordifolia *(*Tc*) itself possesses a bitter, pungent and astringent taste but it acts through the virtue of its madhur (sweet) vipak. The bitter taste is said to improve metabolic activity, even at a cellular level. All the three tastes destroy toxic metabolic products and purify the tissues. Rasayana drugs with madhur vipak are considered to be immunostimulants (13). The root and stem of *Tc *are prescribed in combination with other drugs as an anti-dote to snake bite and scorpion sting (19), dry barks has anti-spasmodic, antipyretic (20), anti-allergic (21), anti-inflammatory (22), anti-leprotic properties (23), Immunomodulatory effect in human Immuno deficiency virus positive patients (24).

In earlier studies, the immunomodulatory activity of *Tc *was studied through different parameters viz. differential leukocyte count (25), phagocytic activity (26), leukocyte migration inhibition (27), delayed type hypersentivity (28), bone marrow cellularity (29), *etc*. Although there are many therapeutic effects, no data is available on the immunomodulatory effect of *Tc *stem (mango plant climber) at cellular level until now. In this context, the objective of the present study was to study the immunomodulator potential of ethanolic extract of *Tc *at cellular and molecular level using male rats.

## Experimental

*Plant material and preparation of extracts*

The stems of *Tc *(mango plant climber) were identified by Dr G. C. Joshi (Botanist), Ranikhet, Uttarakhand, India. A voucher specimen has been preserved in the herbarium at the Pharmacognosy laboratory under the voucher specimen number of PHG/H/2132 for the future reference. Then, the stems of Tc were shade dried and coarsely powdered, extracted in soxhlet apparatus with alcohol for 32 h. The extract was then concentrated to dryness under reduced pressure using rotary evaporator at 45°C and preserved in dessicator for further use.

*Animals*

Male Wister rats weighing 150-180 g were procured from Laboratory Animals Resources, Division of Animal Genetics, Indian Veterinary Research Institute (IVRI), Izatnager, Bairelly, India, Reg No. CPC-196 and acclimatized to laboratory condition at Animal House, IFTM, Moradabad at room temperature with a 12 h/12 h light/dark cycle and relative humidity (60 ± 5%) and had free access to standard pellet diet; water was provided *ad libitum*. The Institutional Animal Ethical Committee reviewed the animal protocol prior to the experiment. All rats were treated in accordance with the guideline for the Care and Use of Laboratory Animals (NIH Publication No. 86-23, revised 1985) with the permission of Institute Animal Ethical Committee (Proposal No. 11). All experiments were carried out with strict adherence to ethical guidelines and were conducted as approved protocol by the Institutional Animal Ethics Committee (IAEC) and as Indian norms laid down by the Committee for the Purpose of Control and Supervision of Experiments on Animals (CPCSEA), New Delhi; vide approval number CPCSEA/IAEC/837/AC/04.

*Animal grouping*

For experimental procedure, male rats were divided into four groups of each six as below: Group I: Negative control: Rats treated with 2 mL of 1% gum acacia solution in distilled water. Group II: Positive control: Sensitized rats (through administrating 1×10^8^ SRBCs, IP) treated with 1% gum acacia solution orally. Group III: Rats treated with cyclophosphamide 100 mg/Kg/p.o. Group IV: Sensitized rats treated with *Tc *ethanolic extract 100 mg/Kg/p.o.

The administrations of drugs were in the following regimens:

a) Four days prior to the sensitization (days -3, -2, -1, 0)

b) Seven days after the sensitization (days +1, +2, +3, +4, +5, +6, +7)

*Preparation of sheep red blood cells (SRBC)*

Blood of healthy sheep was collected from local butcher house and mixed with sterile Alsever’s solution (1 : 1). Then, it was thoroughly mixed and centrifuged at 3000 rpm for 5 min. Supernatant was discarded and SRBC pellets were washed twice with sterilized phosphate buffer saline (pH = 7.2). Then, the SRBC pellets were prepared in phosphate buffer saline (pH = 7.2), total SRBC was counted using Neubauer chamber and finally 1×10^8^ SRBCs (0.5 mL) were injected intraperitoneally for sensitization and challenging the rats (30).

*Immunological activity*

The immunostimulating activity was examined through the selection of *in-vivo *and *in-vitro *immunological tests. Six hours after the last dose, the animals were sacrificed and their livers, pineal glands, spleens, kidneys, bladders and adrenal glands were separated and the following parameters were determined.

*Estimation of liver mitochondrial antioxidant marker enzymes*

For the isolation of mitochondria, 1 g of liver tissue was weighed and homogenized with 5 mL of 0.35 M sucrose buffer (pH = 7.0) at 4°C and centrifuged at 10,000 g for 5 min. The supernatant was discarded and resultant mitochondrial pellets were suspended in a mixture of 1 mL of 10 mM Tris-HCl (pH = 7.4) solution and 0.2 mL of 1 mM EDTA, finally volume was made up to 2 mL with 0.25 M sucrose solution (31). This solution was used for determination of various mitochondrial antioxidant enzymes.

*Estimation of lipid peroxidation (LPO) by homogenization of liver tissue*

After sacrificing the animals, the liver was isolated, weighed, washed with chilled ice-cold sterile 0.9% NaCl solution. Then, the tissue homogenates were prepared in a ratio of 1 g of wet tissue to 9 mL of 1.15% KCl by using a Teflon Potter-Elvehjem homogenizer (32).

*Assay procedure*

A volume of 0.2 mL tissue homogenate was added to the mixture of 0.2 mL of 1% SDS, 1.5 mL of 20% acetic acid solution adjusted to the pH of 3.5 with NaOH, and 1.5 mL of 0.8% aqueous solution of thiobabituric acid. The mixture was made to 4.0 mL with distilled water and then heated in an oil bath at 95°C for 60 min using a glass ball as a condenser. After cooling in tap water, 1.0 mL of distilled water, and 5.0 mL of the mixture of *n*-butanol and pyridine (15:1 v/v) were added to the mixture and shaken vigorously. Then, it was centrifuged at 4000 rpm for 10 min, the organic layer was taken and the absorbance at 532 nm was measured. 1, 1, 3, 3- tetramethoxypropane (TMP) was used as a standard and the level of LPO was expressed as nmol of malondialdehyde (MDA) (33).

*Reduced glutathione (GSH)*

Mitochondrial GSH was estimated through adding 0.2 mL of mitochondrial enzyme solution to 1.8 mL distilled water followed by the addition of 3.0 mL precipitating mixture (0.0501 g metaphosphoric acid, 0.006 g EDTA and 0.9 gm NaCl in 3 mL distilled water). It was centrifuged at 5000 g for 5 min, 1.0 mL of the supernatant was collected and 1.5 mL of the phosphate solution was added to it and then, 0.5 mL of 5,5’-Dithio-Bis (2-nitrobenzoic acid) (DTNB) reagent was added. The optical density was measured at 412 nm spectrophotometrically (Schimadzu 1601). The absorbance of reduced GSH was noted and represented as percentage μMol/gm Hb (34).

*Catalase (CAT)*

A volume of 0.2 mL mitochondrial enzyme solution was added to a cuvette containing 2.0 mL of phosphate buffer (pH = 7.0) and 1.0 mL of 30 mM H_2_O_2_. Catalase activity was measured at 240 nm for 1 min using spectrophotometer. The molar extinction coefficient of H_2_O_2_, 43.6 M cm^-1^, was used to determine the catalase activity. One unit of activity is equal to 1 mmol of H2O2 degraded per minute and is expressed as units/mg of protein (35).

*Superoxide dismutase (SOD)*

Fifteen μL of the mitochondrial enzyme solution was added to a mixture of 0.5 mL of 75 mM Tris-HCl buffer (pH = 8.2), 1 mL of 30 mM EDTA and 1 mL of 2 mM pyrogallol. The absorbance was recorded at 420 nm for 3 min using spectrophotometer. One unit of enzyme activity is calculated as the 50% inhibition of pyrogallol autooxidation rate as determined through the change of absorbance/min at 420 nm. The activity of SOD is expressed as units/mg protein (36).

*SDS-PAGE*

Protein fractions were isolated from the pineal glands of animals from each group and subjected to the SDS-PAGE analysis for the detection of melatonin secretion. Therefore, the resolving gel mixture and the stacking gel mixtures used were 12% and 5% respectively. The gels were stained using Coomassie Brillant Blue R-250 solution for 4 h. Further gels were kept in destaining solution for 8 h until the background gets colourless. Finally, after taking the photographs, the gels were stored in distilled water containing 20% glycerol (37).

*Lymphocyte proliferation assay*

Splenocyte single cell suspension was prepared through up-downing 4 mL of RPMI-1640 in spleen and after omitting RBCs using 0.75% NH_4_Cl in Tris buffer (0.02%, pH = 7.2) (adding 6 mL buffer to 2 mL cell suspension after 3 min of centrifugation at 1000 g for 2 min). The concentration was adjusted to 2×10^6^ Cells/mL in RPMI-1640 supplemented by 10% fetal calf serum, 2 mM L-Glutamine, 25 mM (4-(2-hydroxyethyl)-1-piperazineethanesulfonic acid) (HEPES). A hundred μL of diluted cell suspension was dispensed into a 96-well flat bottom culture plate. Mitogen phytohemmaglutinin A (PHA) was added at 5 μg/mL of final concentration to each well and the volume was adjusted to 0.2 mL. After incubating for 72 h at 37ºC and 5% CO_2_ humid atmosphere air, the cell proliferation was determined using MTT assay method (37). The 10% of (3-(4, 5-dimethyl-2-thiazolyl) 2,5-diphenyl-2H-tetrazolium) (MTT) (5 mg/mL) was added to each well and the plates were incubated at 37ºC in CO_2_ humid atmosphere for 4 h. The blue formazan precipitate was dissolved in acidic isopropanol and its optical density was measured at 570 nm using Elisa Reader. The stimulation index was calculated as follows (39):

Stimulation index = Optical density of stimulated cells/Optical density of unstimulated cells.

*Real-time PCR for the determination of IL-2, IL-10 and TNF-α expression in spleen cells*

Spleen cell suspensions were prepared and used for the determination of cytokines such as IL-2, IL-10 and TNF-*α. *The sequences of primers used in RT-PCR are given in Table 1.

**Table 1:** The sequences of primers used in RT-PCR.

**Primer**	**Sequence (5’ to 3’)**	**Length (bases)**
**IL-10Rat-F**	ACCAGCTGGACAACATACTGCTGA	24
**IL-10Rat-R**	CCTTGATTTCTGGGCCATGGTTCT	24
**IL-2Rat-F**	CTGCAGCGTGTGTTGGATTTGACT	24
**IL-2Rat-R**	TTGCTGGCTCATCATCGAATTGGC	24
**BactRat-F**	TGAGAGGGAAATCGTGCGTGACAT	24
**BactRat-R**	ACCGCTCATTGCCGATAGTGATGA	24
**TNF Rat-F**	CTGGCCAATGGCATGGATCTCAAA	24
**TNF Rat-R**	ATGAAATGGCAAATCGGCTGACGG	24
**RT-BGF**	CATGTTTGTGATGGGCGTGAACCA	24
**RT-BGR**	TAAGTCCCTCCACGATGCCAAAGT	24

*RNA isolation*

Trizol LS reagent (Invitrogen cat No. 10296-010) was used for the RNA isolation and the method prescribed by the manufacturer was followed. Briefly, spleen cells were washed with PBS lysed through adding Trizol LS reagent onto them and repeated pipetting. The organic and aqueous phases were separated with the help of chloroform and centrifugation. Total RNA from the aqueous phase was precipitated with isopropyl alcohol, washed in ethanol, air dried and diluted in nuclease free water.

*cDNA preparation*

Revert Aid M-MuLV Reverse Transcriptase (Fermentas cat No. EP0441) was used for this purpose. About 100 ng of RNA and 0.5 μg of oligo dT (13-19) primer (Invitrogen cat No. 18418-012) were mixed and snap chilled. Four μL of 5× reaction buffer, 20 U of RNase inhibitor, 2 μL of 10 mM dNTP and 200 U of Revert Aid M-MuLV Reverse Transcriptase were added to this mixture. The final reaction volume was kept 20 μL. The reaction mixture was incubated at 42°C for 1 h. The reaction was then stopped through heating the mixture at 70ºC for 10 min. The cDNA was divided into aliquots of suitable size and stored at -20°C (40).

*Real-time PCR*

Brilliant SYBR Green QPCR Core Reagent Kit (Stratagene cat No. 600546) was used for this purpose. One μL of cDNA was added to 11 μL of nuclease free water, 2.5 μL of 10×core PCR buffer, 1.25 μL of 50 mM MgCl_2_ solution, 1 μL of 20 mM dNTP mix, 1 μL (12.5 pM/μL) of forward primer, 1 μL (12.5 nM/μl) of reverse primer, 4 μL of 50% glycerol solution, 0.75 μL of 100% DMSO, 1.25 μL of 1:3000 diluted SYBR Green I dye and 0.25 μL (1.25 U) of SureStart Taq DNA polymerase. Real-time PCR was carried out in M×3000p (Stratagene). The thermal profile used for this was as follows: 95ºC for 10 min then 40 cycles of 95ºC for 30 sec, 64ºC for 30 sec and 72°C for 30 sec with fluorescence recording at the end of each cycle, followed by denaturation of products from 55ºC to 95ºC with fluorescence recording throughout the step. Fold changes in target transcript levels were determined using reported method (40), considering E_target_ = E_ref_ = 2. The GAPDH gene was used as a reference.

*Statistical analysis*

The results were expressed as mean ± SD and the statistical evaluation of the data was done using the prism software (version 5.00) and Student’s t-test. P-values less than 0.05 were considered as significant.

## Results and Discussion

*Liver mitochondrial enzyme estimation*

Table 2 shows the increased lipid peroxidation level in liver (98.38 ± 1.14 MMDA/g Hb) in SRBC-treated group as compared to the vehicle-treated rat (83.85 ± 2.12 MMDA/g Hb). Furthermore, cyclophosphamide-treated rats showed slight increase (90.42 ± 2.13 MMDA/g Hb) in the lipid peroxidation level in the liver and after the treatment of *Tc *ethanolic extract, the elevated lipid peroxidation was decreased to 62.52 ± 1.13 ± 1.16 MMDA/g Hb.

**Table 2:** Effect of *Tc *on rat’s liver mitochondrial enzymes

**Treatment group**	**Liver Mitochondrial Enzymes**			
	**LPO (MMDA/g Hb)**	**GSH (μMol/g Hb)**	**SOD (units/mg protein)**	**Catalase (units/mg protein)**
**Vehicle**	83.85 ± 2.12	4.22 ± 0.21	22.22 ± 0.10	271.31 ± 3.2
**SRBC sensitized**	98.38 ± 1.14	2.13 ± 0.18	19.48 ± 0.16	252.22 ± 2.5
**Cyclophosphamide**	90.42 ± 2.13	2.31 ± 0.38	20.38 ± 0.18	256.29 ± 3.8
***Tc***	62.52 ± 1.13*	4.82 ± 0.31*	25.32 ± 0.14*	278.41 ± 9.2*

*p < 0.05 when compared to SRBC sensitized and cyclophosphamide-treated groups

The levels of reduced glutathione, superoxide dismutase and catalase were decreased through the SRBC sensitization and were increased via the treatment with *Tc *ethanolic extract. The levels of reduced glutathione, superoxide dismutase and catalase, treated by SRBC sensitization and *Tc *ethanolic extract, were 2.13 ± 0.18 and 4.82 ± 0.31 μMol/g Hb, 19.48 ± 0.16 and 25.32 ± 0.21 units/mg protein and 252.22 ± 2.5 and 278.41 ± 9.2 units/mg protein respectively. Cyclophosphamide showed immunosupression through increasing the lipid peroxidation level and decreasing the level of reduced glutathione, superoxide dismutase and catalase as compared with the control group.

The oxidation of membrane’s lipid molecules damages the development of several physiological and pathological disorders. The inhibition of lipid peroxidation by any means is the best way to avoid these disorders in the body (41). Present study showed the effect of *Tc *ethanolic extract on lipid peroxidation which was inhibitorier as compared with SRBC and cyclophosphamide-treated groups.

Glutathione is the major endogenous antioxidant produced by the cells, participating directly in the neutralization of free radicals and reactive oxygen compounds, as well as maintaining the exogenous antioxidants such as vitamins C and E in their reduced (active) forms. Through the direct conjugation, it detoxifies many xenobiotics (foreign compounds) and carcinogens, both organic and inorganic. It is essential for the immune system to exert its full potential viz. modulating the antigen presentation to lymphocytes, and thereby, influencing the cytokine production; enhancing the proliferation of lymphocytes thereby increasing magnitude of response; enhancing the killing activity of cytotoxic T-cells and natural killer cells and regulating apoptosis thereby maintaining the control of immune response. Glutathione plays a fundamental role in numerous metabolic and biochemical reactions such as DNA synthesis and repair, protein synthesis, prostaglandin synthesis, amino acid transport and enzyme activation. Thus, every system in the body can be affected through the state of glutathione system, especially the immune system, the nervous system, the gastrointestinal system and the lungs (42-45).

Superoxide dismutase induces the activation of endogenous system of the antioxidant defenses which fights against free radicals (46). It is known that superoxide dismutase plays an important role in the detoxification of superoxide anion and H_2_O_2_, and thereby promotes the cell against the free radical-induced damage (47).

Catalase is an enzymatic antioxidant and helps in neutralizing the toxic effect of H_2_O_2_. Hydrogen peroxide is not reactive enough to cause a chain of lipid peroxodation reactions, but its combination with superoxide radical produces hydroxyl radicals that are highly reactive and thus initiates the lipid peroxidation reaction (48). This lipid peroxidation damages the cell membrane resulting into the development of several physiological and pathological disorders. Catalase prevents this through the conversion of hydrogen peroxide to water and non-reactive oxygen species and thus, prevents from the generation of hydroxyl radical and protect the cell from the oxidative damage (49, 50). Our studies showed distinct increase in the level of glutathione, superoxide dismutase and catalase in case of *Tc*-treated groups as compared to SRBC and cyclophosphamide-treated groups. Therefore, *Tc *has antioxidant activity as well as immunomodulator property through protecting the cells from the oxidative damage.

*Separation of pineal proteins through SDS-PAGE*

**Figure 1:**
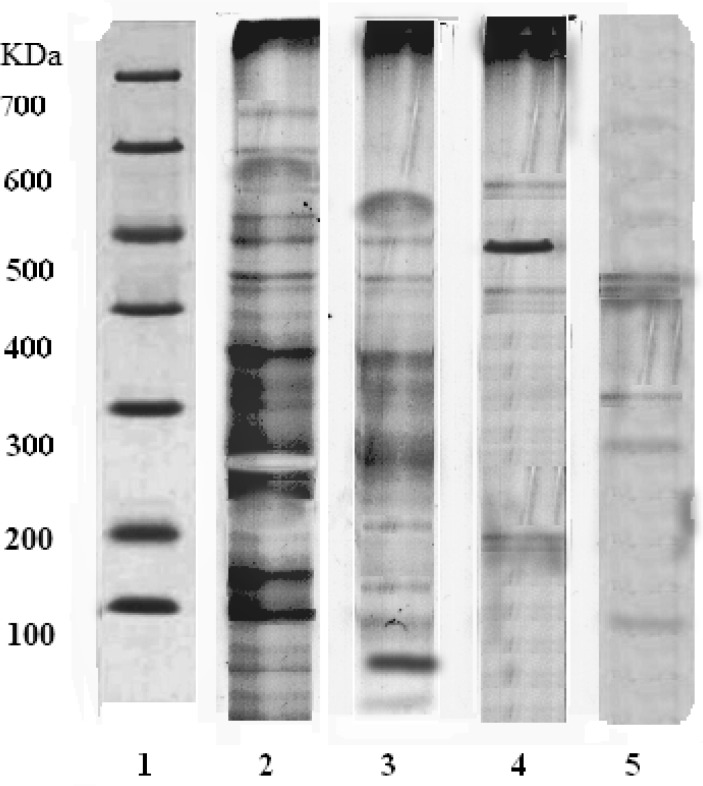
SDS-PAGE of rat pineal gland. Lane 1: Indicates the protein marker of a standard; Lane 2: *Tc *ethanolic extract treatment shows thick band of protein expression (248 kDa) indicating the increased secretion of melatonin via pineal gland due to the immunomodulatory action; Lane 3: Control group; Lane 4: SRBC sensitized group; Lane 5: Immunosuppressant action of cyclophosphamide

A thick band at 248 kDa appeared in the group treated with *Tc *ethanolic extract indicating higher secretion of melatonin (Mol. Wt. 248 kDa) (lane 2). In SRBC-sensitized group, the expression of proteins (lane 4) was less than that of control group (lane 3). The immunosuppressant cyclophosphamide showed the least expression of proteins as compared to other groups (lane 5). The increased expression of melatonin in lane 2 confirms the immunomodulator activity of *Tc *(Figure 1).

Melatonin plays an important role as immunomodulatory (51-53) properties, as well as many other physiological functions (54). Melatonin was reported to be an integral part of the immune system, through exerting direct and/or indirect stimulatory effect on both cellular and humoral immunity (51). The present study showed that *Tc *treatment shows the thick band of protein expression indicating the increased production of the melatonin as compared to SRBC sensitized, vehicle and cyclophosphamide-treated groups. Hence, *Tc *exhibits immunostimulant action through enhancing melatonin secretion in pineal gland.

*Lymphocyte proliferation assay*

The administration of *Tc*-ethanolic extract increased the spleen lymphocytes proliferation (1.428 ± 0.361) as compared to the vehicle-treated group (0.787 ± 0.256) and cyclophosphamide-treated rats (0.832 ± 0.471) (Table 3). Phytohaemagglutinin used as a standard shows proliferation of lymphocytes in all groups.

**Table 3:** Proliferation of rat spleen lymphocytes in response to antigen prepared from *Tc *ethanolic extract using MTT dye reduction test

**Treatment**	**Stimulation index**	**Phytohaemagglutinin-stimulated**
Vehicle	0.787 ± 0.256	1.222 ± 0.841
Cyclophosphamide	0.832 ± 0.471	0.936 ± 0.412
*Tc*	1.428 ± 0.361*	1.386 ± 0.361*

*p < 0.05 when compared with control and cyclophosphamide-treated group

The earlier works have revealed that drugs having immunomodulatory activity show enhanced proliferation of splenocytes (55). In present study, *Tc *was found to significantly enhance the proliferation of splenocytes as compared with control and cyclophosphamide treatment. The proliferation of lymphocytes indicated the increase in the number of B and T cells, which release cytokines and growth factors that regulate other immune cells and secretion of antibodies in the blood (56).

*Comparison of IL-2, IL-10 and TNF-α gene expression in rat spleen mRNA*

Measuring and assessing cytokine profile provides a useful method for the accurate study of cytokine such as IL-2, IL-10 and TNF-*α *and immunomodulatory effect was investigated through RT-PCR. *Tc *treatment showed more fold changes in IL-2, IL-10 and TNF-*α *mRNA (17.63, 42.52 and 5.39 respectively) levels as compared to the vehicle, cyclophosphamide and SRBC sensitized groups as shown in Table 4. Increased expression of fold change in different mRNA levels using *Tc c*onfirms the immunostimulant action.

**Table 4:** Effect of different treatments on IL-2, IL-10 and TNF-α gene expression

**Treatment**	**Fold change in IL-2, IL-10 and TNF- α mRNA level**		
	**IL-2**	**IL-10**	**TNF-α**
Vehicle	1.00	1.00	1.00
Cyclophosphamide	6.41	8.63	-1.78
*Tc*	17.63	42.52	5.39
SRBC sensitized	-2.87	22.63	1.18

Cytokines are released by living cells of the host in a highly regulated fashion to regulate the cell functions via specific receptors (57) which participate in the control of all immunologically relevant events, whether they concern the activation, differentiation, maturation, proliferation, apoptosis, or acquisition of effectors’ functions. Cytokines influence the quantitative, as well as the qualitative outcome of all the immune responses (58). In our study, after the administration of ethanolic extract of *Tc *in male Wister rats, significant immunomodulatory activities were shown through fold changes in IL-2, IL-10 and TNF-*α *mRNA levels. IL-2 shows immunomodulatory activities by T and B lymphocytes proliferation, natural killer cell activation (59, 60) and also increasing in the immunoglobulin G production whereas IL-10 promotes elevation of Th_2_ cells (58, 60) and TNF-*α *modulate the cytokine gene expression (59). Hence, the immunomodulatory activity of *Tc *may be due to the above mechanism(s).

## Conclusion

From the above results, it can be concluded that *Tc *stem (mango plant climber) has potent immunomodulatory activity and the effect may be due to the specific chemical constituents or the synergistic effect of chemical compounds of the drug.
